# Immune Adjuvant Activity of Pre-Resectional Radiofrequency Ablation Protects against Local and Systemic Recurrence in Aggressive Murine Colorectal Cancer

**DOI:** 10.1371/journal.pone.0143370

**Published:** 2015-11-23

**Authors:** Fumito Ito, Amy W. Ku, Mark J. Bucsek, Jason B. Muhitch, Trupti Vardam-Kaur, Minhyung Kim, Daniel T. Fisher, Marta Camoriano, Thaer Khoury, Joseph J. Skitzki, Sandra O. Gollnick, Sharon S. Evans

**Affiliations:** 1 Department of Immunology, Roswell Park Cancer Institute, Buffalo, New York, United States of America; 2 Department of Surgical Oncology, Roswell Park Cancer Institute, Buffalo, New York, United States of America; 3 Department of Surgery, University of Michigan, Ann Arbor, Michigan, United States of America; 4 Department of Urology, Roswell Park Cancer Institute, Buffalo, New York, United States of America; 5 Edwin L. Steele Laboratory, Department of Radiation Oncology, Massachusetts General Hospital, Harvard Medical School, Boston, Massachusetts, United States of America; 6 Department of Pathology, Roswell Park Cancer Institute, Buffalo, New York, United States of America; 7 Department of Cell Stress Biology, Roswell Park Cancer Institute, Buffalo, New York, United States of America; Istituto Superiore di Sanità, ITALY

## Abstract

**Purpose:**

While surgical resection is a cornerstone of cancer treatment, local and distant recurrences continue to adversely affect outcome in a significant proportion of patients. Evidence that an alternative debulking strategy involving radiofrequency ablation (RFA) induces antitumor immunity prompted the current investigation of the efficacy of performing RFA prior to surgical resection (pre-resectional RFA) in a preclinical mouse model.

**Experimental Design:**

Therapeutic efficacy and systemic immune responses were assessed following pre-resectional RFA treatment of murine CT26 colon adenocarcinoma.

**Results:**

Treatment with pre-resectional RFA significantly delayed tumor growth and improved overall survival compared to sham surgery, RFA, or resection alone. Mice in the pre-resectional RFA group that achieved a complete response demonstrated durable antitumor immunity upon tumor re-challenge. Failure to achieve a therapeutic benefit in immunodeficient mice confirmed that tumor control by pre-resectional RFA depends on an intact adaptive immune response rather than changes in physical parameters that make ablated tumors more amenable to a complete surgical excision. RFA causes a marked increase in intratumoral CD8^+^ T lymphocyte infiltration, thus substantially enhancing the ratio of CD8^+^ effector T cells: FoxP3^+^ regulatory T cells. Importantly, pre-resectional RFA significantly increases the number of antigen-specific CD8^+^ T cells within the tumor microenvironment and tumor-draining lymph node but had no impact on infiltration by myeloid-derived suppressor cells, M1 macrophages or M2 macrophages at tumor sites or in peripheral lymphoid organs (i.e., spleen). Finally, pre-resectional RFA of primary tumors delayed growth of distant tumors through a mechanism that depends on systemic CD8^+^ T cell-mediated antitumor immunity.

**Conclusion:**

Improved survival and antitumor systemic immunity elicited by pre-resectional RFA support the translational potential of this neoadjuvant treatment for cancer patients with high-risk of local and systemic recurrence.

## Introduction

Surgery remains the frontline therapy and best curative option for cancer patients with resectable disease. The goal of surgical excision is complete removal of tumors with microscopic cancer-free margins. Unfortunately, many patients develop local or systemic relapse despite advances in surgical technique and adjuvant chemotherapy or radiotherapy. In colorectal liver metastasis, for example, the relapse rate is up to 60% in patients undergoing a potentially curative resection, presumably due to microscopic residual disease or micrometastases present at the time of surgery [1–3]. Evidence of a high rate of recurrence and metastatic dissemination in patients post-surgery further suggests that although systemic antitumor immunity is evident in advanced cancer patients, the steady-state immune response is insufficient to provide adequate tumor control [4]. By the time tumors are detectable they have already developed mechanisms to escape from immunologic recognition and elimination [4]. Thus, resected tumor specimens often show sparse infiltration by CD3^+^ T lymphocytes, including CD8^+^ cytotoxic effector T cells, which correlates with poor prognosis in multiple histological tumor types including colorectal cancer [5–7].

*In situ* thermal ablation techniques, such as radiofrequency ablation (RFA), have emerged as promising treatment options for unresectable solid malignant tumors [8, 9]. RFA is approved by the US Food and Drug Administration (FDA) for ablation of primary and metastatic tumors, and has been applied in the treatment of a variety of neoplasms including liver, colorectal, lung, prostate, kidney, breast, brain, thyroid, melanoma, and bone tumors [9]. In the management of colorectal cancer liver metastases, RFA has been used most frequently *via* open, laparoscopic, or image-guided percutaneous approaches and can be performed under general or local anesthesia with minimal complications [8, 9]. RFA is also used in combination with surgical resection in patients with multiple liver lesions in which complete resection is not feasible due to tumor proximity to vital structures (e.g., blood vessels or bile ducts) or insufficient parenchyma to support post-hepatectomy function [2, 10].

The energy delivered by RFA causes ionic agitation which is converted by friction into focal high-temperature hyperthermia (≥50°C), thereby inducing irreparable cellular damage and coagulative necrosis [8]. In addition to this direct cytoreductive antitumor activity, RFA has been shown to elicit immunomodulatory effects in preclinical and clinical studies [8, 11]. In contrast to surgical resection, the damaged tumor tissue following RFA remains *in situ* and provides a source of tumor antigens. These antigens are taken up and processed by local dendritic cells (DC) and presented to T lymphocytes in tumor-draining lymph nodes (TdLN), thus leading to the expansion of a tumor-specific CD8^+^ effector T cell pool [8, 12, 13]. Notably, a dense CD3^+^ T cell infiltrate has been reported at tumor sites after RFA, consistent with local antitumor immune reactivity [14, 15]. Further evidence of RFA-induced systemic immunity stems from preclinical and clinical reports of abscopal effects involving spontaneous regression of distant metastatic lesions after primary tumor ablation [16–20]. However, the T cell-dependent antitumor immune response generated by RFA in patients and mouse models appears to be relatively weak [11, 14, 15] and a major problem remains the high rate of local recurrence at the treatment site, especially for tumors >3 cm or close to major vascular networks where blood inflow limits thermal concentration in the target tissue via heat-sink effects [2, 8].

Concerns about tumor regrowth in the transitional zone of sublethal hyperthermia (≤45°C) that surrounds the central necrotic zone have limited the use of RFA mainly to palliation or to patients who cannot tolerate resectional surgery. Here, we tested the hypothesis that local surgical outcome could be improved if RFA was used as a pre-resectional immunogenic strategy to boost antitumor immunity. We report that local and systemic tumor control and overall survival were significantly improved as a result of the induction of a CD8^+^ T cell-mediated adaptive immune response following pre-resectional RFA in a mouse model for colorectal cancer.

## Materials and Methods

### Mice

Female BALB/c and C57BL/6 mice (8-12-week) were from National Cancer Institute or Charles River. Severe combined immunodeficient mice (CbySmn.CB17-Prkdc^scid^/J SCID/BALB/c; Jackson Laboratory) and Clone 4 transgenic mice (CBy.Cg-*Thy1a* Tg(Tcra(C14,TcrbC14)1Shrm/ShrmJ) expressing an α/β T cell receptor specific for influenza hemagglutinin (HA; from Linda Sherman, Scripps Research Institute, La Jolla, CA) were bred in the Roswell Park Department of Laboratory Animal Resources. Mice were maintained in pathogen-free barrier conditions. All animal care and procedures were in accordance with institutional policies for animal health and well-being and approved by the Roswell Park Cancer Institutional Animal Care and Use Committee (IACUC).

### Transplantable tumor models and CD8^+^ T cell depletion

Tumor cells (CT26 colon adenocarcinoma cells and the CT26-HA derivative transfected with a gene encoding hemagglutinin [21, 22] syngeneic to BALB/c or SCID mice; B16.F10 melanoma syngeneic to C56BL/6 mice) were cultured in RPMI 1640 supplemented with 10% heat-inactivated fetal calf serum (Life Technologies, Carlsbad, CA), 2 mM L-glutamine, 100 U/ml penicillin, 50 μg/ml streptomycin, 50 μM β-mercaptoethanol, and G418 (400 μg/ml; for CT26-HA). Tumor cells (10^6^ for CT26 or 3x10^5^ for B16; in 100 μL PBS) were injected s.c. in the flank of syngeneic mice, unless otherwise indicated. Tumor volumes (ranging from 150–500 mm^3^) were not significantly different between assigned groups at the time of initial treatment. Tumor volumes were calculated by determining the length of short (*l*) and long (*L*) diameters (volume = l^2^ x L/2). Experimental end points were reached when tumors exceeded 20 mm in diameter or when mice became moribund and showed signs of lateral recumbancy, cachexia, lack of response to noxious stimuli, or observable weight loss. The ipsilateral inguinal node was identified as the sentinel TdLN by localization of Evans blue dye 1 minute after injection of dye near the s.c. tumor site. To model end stage lung metastasis, 10^5^ CT26 cells were injected in the tail vein 7 days after primary, s.c. tumor injection; 30 days later, CT26 nodules were enumerated by investigators blinded to sample identity in inflated lungs stained with a 15% solution of India ink, and bleached by Fekete’s solution. For depletion of CD8^+^ T cells in vivo, mice were injected i.p. with 400 μg of anti-CD8α mAb (53–6.72, BioXCell) every 7 days starting immediately post-RFA treatment. CD8^+^ T cell depletion was confirmed by flow cytometry using anti-CD8β mAb (YTS156.7.7, Pe-Cy7) (BioLegend); < 2% of CD3^+^ T cells were CD8^+^ after antibody treatment.

### RFA

Mice were anesthetized with inhalational isoflurane gas (4% isoflurane for anesthesia induction; 1.5% for maintenance). Mice were positioned prone on an electricity-conducting grounding pad, and the tumor area was wet with distilled water prior to inserting the StarBurst SDE probe (AngioDynamics) into the center of the tumor. Maintining a probe tip temperature of 90°C for 1 minute using the RFA RITA 1500 generator (AngioDynamics) simulated the clinical setting of tumor recurrence after RFA, as previously described by Johnson et al [23]. Sham RFA was performed by inserting the probe into the tumor without electrical current. For additional control groups RFA was administered in the skin away from the s.c. tumor (i.e., upper back). Mice were recovered on a warming blanket and given analgesic (buprenorphine 0.05mg/kg body weight) s.c. for pain control.

### Surgical resection

Mice were anesthetized with inhalational isoflurane gas and tumors were excised with 1 mm margins around the visible border of the tumor. Fascia was resected together with tumor (*en bloc*) in all cases to minimize recurrence from the deep margin. Pathological evaluation of the tumor/stromal interface at the time of excision in sentinel mice revealed infiltrative tumor in the borders in 40–60% of mice regardless of whether pre-resectional RFA was administered. Incisions were closed with 4–0 Vicryl® suture (Ethicon, Inc., Somerville, NJ) and/or steel wound clips which were removed in 7–10 days. As a surgical sham control, mice underwent a 1 cm skin incision and closure in the contralateral flank without manipulation of the tumor site. In selected experiments, partial resection was performed, classified by detectable macroscopic and microscopic residual disease in which ~50% of the tumor mass was excised. Mice were recovered on a warming blanket and given analgesic (buprenorphine 0.05mg/kg body weight) s.c. for pain control.

### Adoptive transfer of Clone 4 CD8^+^ T cells

Pooled splenocytes and lymph node (LN) cells from Clone 4 T-cell receptor transgenic mice were positively enriched by magnetic cell sorting using a CD8α T cell isolation kit (Miltenyi Biotec, Auburn, CA). Isolated populations were comprised of ~85–90% L-selectin^hi^ CD44^low^ naïve CD8^+^ T cells as determined flow cytometric analysis. Equivalent numbers of Clone 4 CD8^+^ T cells (5–10 x 10^6^) labeled with tracking dye (4 μM TRITC; Molecular Probes) [22, 24] were injected i.v. into individual mice 6 hours after RFA or sham treatment. TdLN and tumor tissues were frozen in optimum cutting temperature compound (Sakura Finetek).

### Serum IFN-γ analysis

Serum was acquired from mice 1 day and 7 days-post RFA or sham treatment via retro-orbital bleeding. Specimens were stored frozen at -80°C until assay by commercial ELISA for IFN-γ (R&D Systems).

### Immunohistochemistry and immunofluorescence histology

For immunohistochemical analysis, serial 9 μm cryostat sections of TdLN or tumor were fixed in acetone, blocked with Superblock Blocking Buffer (Pierce), and stained with rat anti-mouse monoclonal antibodies specific for CD8α (53–6.7; BD Biosciences) [22]. Immune complexes were visualized using the Vectastain Elite ABC and DAB substrate kits (Vector Laboratories). For immunofluorescence histology, 9 μm cryosections were fixed at -20°C in methanol/acetone (3:1), and stained with monoclonal antibodies (anti-mouse PNAd antibody, MECA-79; anti-mouse CD31 antibody, MEC 13.3, BD Biosciences) and fluorochrome-conjugated secondary antibody (Jackson ImmunoResearch) as described [22, 24]. Digital images of ≥10 randomly selected fields (unit area = 0.34 mm^2^ per field) were captured by observers blinded to sample identity using an Olympus BX50 upright fluorescence microscope equipped with a SPOT RT camera (Spectra Services). The number of adoptively transferred cells located within the parenchyma of TdLN or tumors (i.e., designated by location of fluorescent-labeled T cells outside PNAd^+^ or CD31^+^ vessels, respectively) was quantified with ImageJ software as described [21, 24, 25].

### Flow cytometry

CT26 tumors from 1,100–1,700 mm^3^ were used for RFA treatment in order to have sufficient residual tumor tissue to analyze by flow cytometry. Tumors or spleens were disaggregated (Medimachine, Becton Dickinson, Franklin Lakes, NJ) and phenotypic analysis was performed using multiparameter flow cytometry following staining with monoclonal antibodies: anti-CD8α (53–6.7, Pe-Cy7), anti-CD4 (RM4-5, PerCP or V450), anti-CD25 (PC61, APC), anti-L-selectin (Mel-14, APC or PE), anti-CD44 (IM7, FITC), anti-CD3 (17A2, FITC), anti-CD11b (M1/70, Pe-Cy7), anti-Gr-1 (RB6-8C5, APC), anti-F4/80 (T45-2342, PE), anti-MHC II (2G9, FITC), anti-CD45 (30-F11, BUV395) (BD Biosciences); anti-FoxP3 (FJK-16s, PE) (eBioscience); anti-CD8β (YTS156.7.7, Pe-Cy7), and anti-CD206 (C068C2, BV421) (BioLegend). Flow cytometric analysis was conducted using an LSR II or LSRFortessa with FACSDiva software (BD Biosciences) and WinList processing program (Verity Software House).

### Statistical analysis

Statistical significance of tumor growth was determined by 2-way ANOVA for repeated measures. Survival was analyzed with the Kaplan-Meier method and groups were compared using log-rank test or by the Gehan-Breslow-Wilcoxon test. All other group differences were evaluated by 2-tailed unpaired Student's *t*-test. Data are presented as mean ± SEM and *P* <0.05 was considered statistically significant.

## Results

### Pre-resectional RFA improves local tumor control and survival

**Fig 1:**
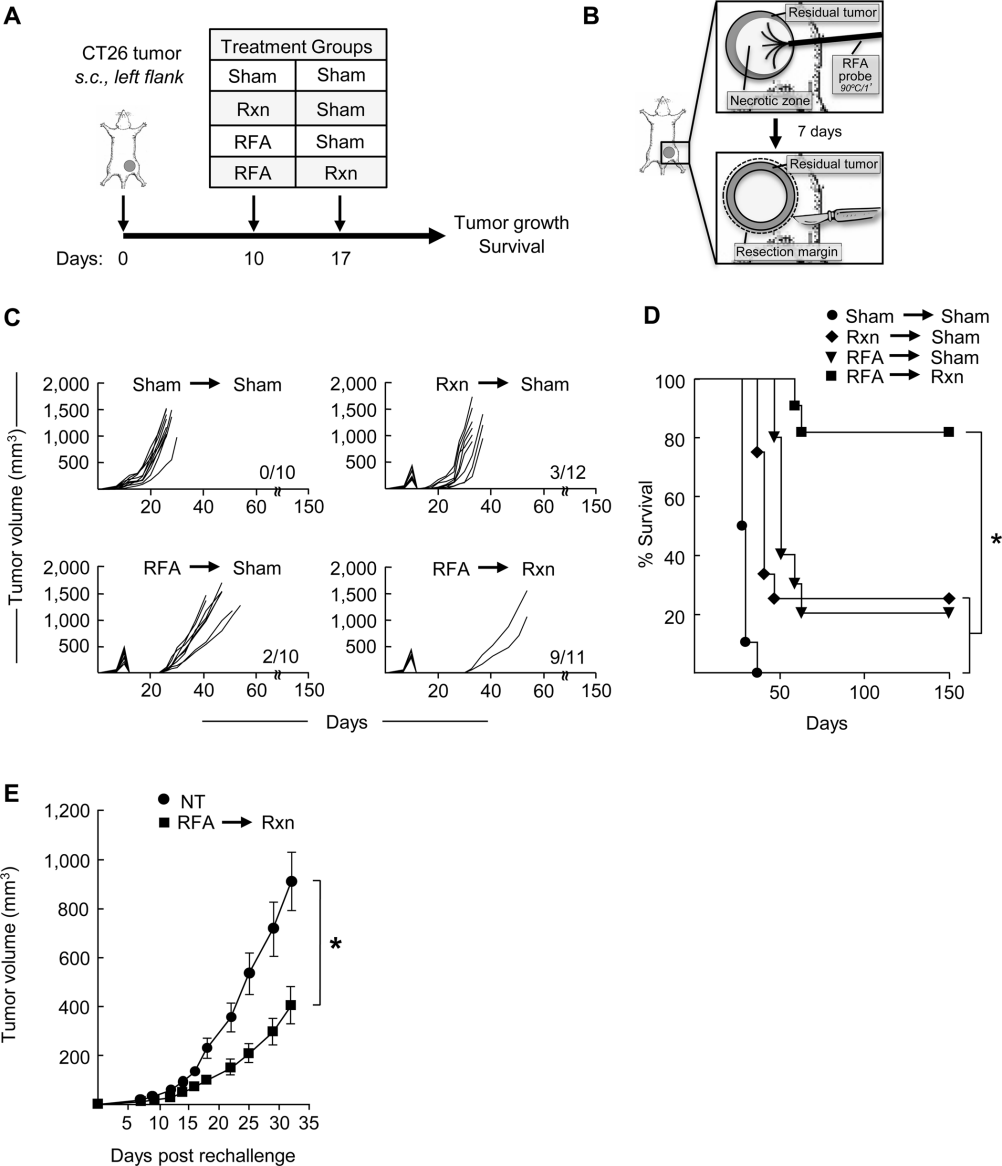
Pre-resectional RFA improves local CT26 tumor control and survival. **(A)** Time schedule outlining the different treatments used. BALB/c mice (10–12 mice/group) were injected s.c. with 10^6^ CT26 cells into the left flank on day 0. Treatments were performed 10 days after tumor innoculation. Primary tumors were treated as indicated with sham surgery, resection (Rxn), or RFA on days 10 or 17. **(B)** Schematic showing sequence of administration of RFA (90°C for 1 minute) 7 days prior to surgical resection. Tumor growth curves **(C)** and survival curves **(D)** of mice bearing CT26 tumors in different treatment groups. In **(C)**, the number of long-term survivors without tumor recurrence at 150 days is indicated for each experimental group; for **(D)** *P < 0.001 for pre-resectional RFA group compared to all other groups. **(E)** Surviving mice (n = 9) in the pre-resectional RFA group were rechallenged with 10^6^ CT26 cells into the contralateral (right) flank on day 150. Tumor growth curves are depicted in which *T* = 0 corresponds to the time of injection of secondary tumors. As a control, tumor growth was monitored following inoculation of the same tumor cell dose into non-tumor (NT)-experienced naive BALB/c mice (n = 10); *P < 0.005 as determined by Kaplan-Meier analysis. One representative experiment of ≥ three independent experiments is shown.

To determine whether RFA treatment prior to surgical resection could decrease local recurrence and improve survival, we used a mouse model for CT26 colon adenocarcinoma to simulate the clinical setting where RFA provides initial tumor control but is often followed by local recurrence. In pilot studies we optimized the size of the tumor at the time of ablation together with the temperature and duration of RFA. For these studies s.c. CT26 tumors of approximately equivalent size (~150–500 mm^3^ in volume) were treated with RFA (1 minute of ablation at 90°C) on day 10 after implantation. Resection of the same lesion was performed on day 17, as indicated in the schematic shown in Fig 1A and 1B. We elected to use this 7-day interval between RFA and surgery to allow sufficient time for a primary antitumor immune response to be generated following ablation [11, 13, 26]. Control mice had resection or RFA only on day 10 followed by sham surgery (outside tumor region) on day 17, or sham surgeries on both day 10 and 17 (Fig 1A).

Using this experimental plan we found that treatment of CT26 with RFA alone resulted in 60–80% necrosis of individual tumor lesions followed by ≥ 75% tumor recurrence if no additional therapy was given (Fig 1C). Similarly, surgical excision alone was associated with a ≥ 75% local recurrence rate. In contrast, the combination of RFA followed by resection markedly delayed tumor outgrowth compared with single treatment groups or sham excision control groups (Fig 1C). Notably, tumor recurrence was not detected for more than 150 days in ~80% (9 of 11 mice) of mice treated with the combination of RFA and subsequent surgery. RFA followed by resection further showed significantly improved survival compared with all other groups in this lethal CT26 tumor model (Fig 1D). In this regard, RFA followed by resection resulted in ~80% local recurrence-free survival compared with ~ 20% survival following single treatment with either RFA or resection alone.

Additional experiments excluded the possibility that the observed therapeutic benefit of combined RFA and resection could be attributed to *(1)* differences in the total time the host was exposed to tumor antigens in different treatment groups (i.e., 10 days for the resection followed by sham surgery group versus 17 days for RFA + resection group), *(2)* more effective surgery because of initial tumor debulking during pre-resectional RFA, or *(3)* inflammation triggered by probe insertion (S1A Fig). For these experiments, we observed no improvement in CT26 tumor growth delay or survival if initial tumor-debulking was achieved on day 10 by partial resection (i.e., 50% reduction of tumor mass) followed by excisional resection on day 17 or after sham RFA in which the ablation probe was inserted into tumors without electrical current (S1B and S1C Fig). Additional studies in B16 melanoma in syngeneic C57BL/6 mice (S2A–S2C Fig) established that RFA in the neoadjuvant setting prior to surgery has a similar therapeutic benefit as observed for CT26 colon tumors. Thus, the protective activities of pre-resectional RFA are not restricted to a particular histological tumor type.

A hallmark of adaptive immunity is the development of immunological memory defined by the ability of previous exposure to tumor antigens to trigger a recall response upon re-exposure. Therefore, we investigated whether mice surviving after combined RFA and resection of CT26 tumors developed antitumor reactivity by rechallenging long-term survivors (i.e., 150 days post-primary tumor implantation) with s.c. CT26 tumor in the contralateral flank. Age-matched, untreated non-tumor (NT)-experienced naïve mice were used as controls. As shown in Fig 1E, a significant delay in the outgrowth of CT26 tumor was observed in rechallenged mice that had been previously treated with RFA followed by surgical excision. Taken together, these data are consistent with the development of an adaptive immune response after pre-resectional RFA that reduces local recurrence and improves survival by promoting long-lasting, systemic immunity.

### Survival benefit of pre-resectional RFA depends on adaptive immunity

**Fig 2:**
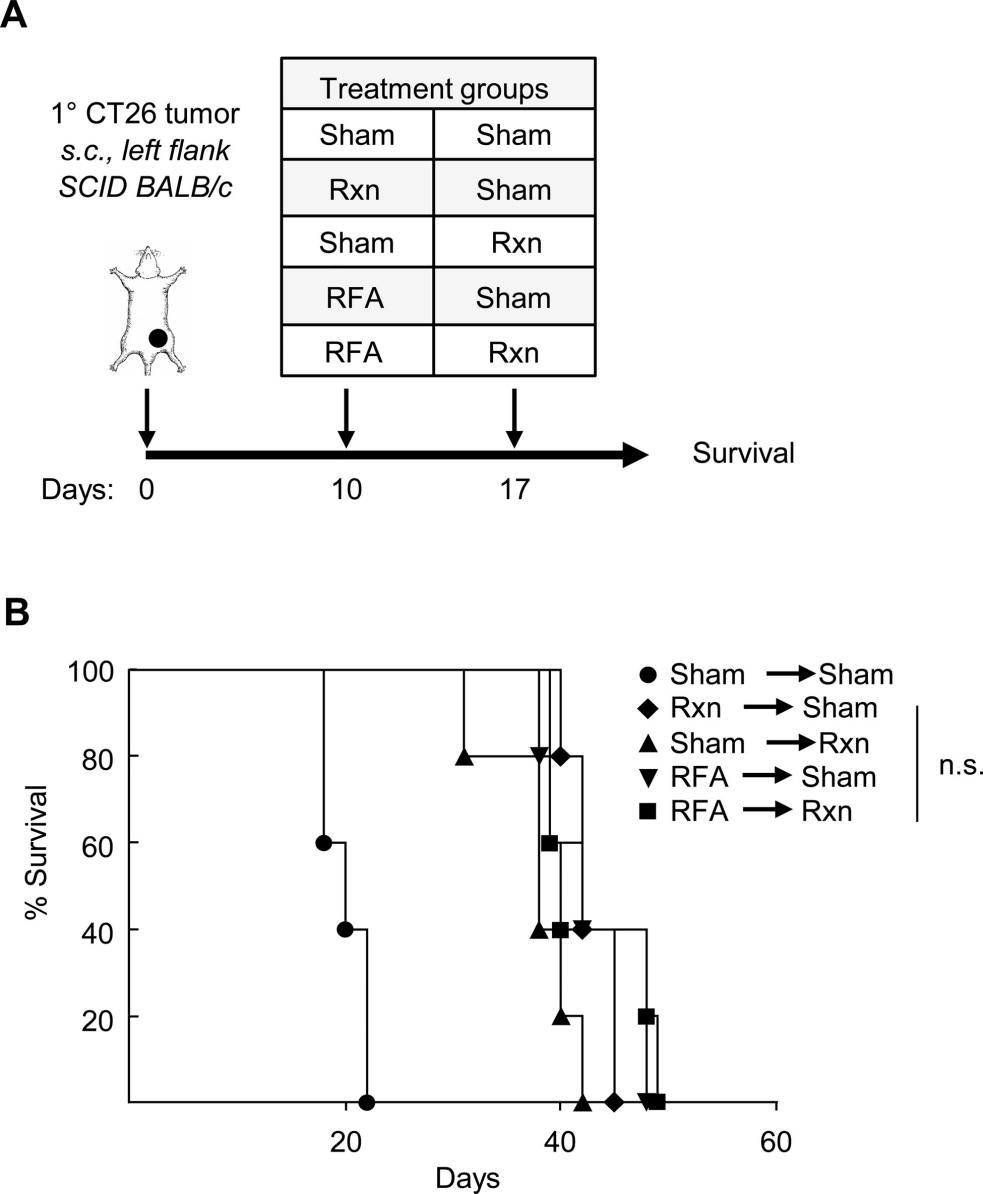
Survival benefit of pre-resectional RFA depends on adaptive immunity. **(A)** Time schedule outlining treatment groups. SCID mice (5 mice/group) were implanted s.c. with 10^6^ CT26 cells into the left flank on day 0. Treatment groups incuded sham surgery, resection (Rxn), or RFA on days 10 or 17. **(B)** Survival curves are representative of two independent experiments; n.s., not significant as determined by Kaplan-Meier analysis.

To formally investigate whether RFA activation of a tumor-specific adaptive immune response prior to surgical excision is causal to limiting tumor growth we examined the effects of this combined therapeutic regimen on CT26 tumor progression in severe combined immunodeficient (SCID) mice that lack functional T cells and B cells (Fig 2A). Survival was used as the primary endpoint for determining efficacy since analysis of primary tumor volume was not informative in immunodeficient mice which frequently succumbed to disseminated disease (i.e., in the peritoneum) irrespective of primary tumor size or the type of therapy. In sharp contrast to the survival benefit observed in immunocompetent mice (Fig 1D), we failed to detect any survival advantage in SCID mice treated with combined RFA and resection when compared with either treatment alone (Fig 2B). This dependence on the adaptive immune response provides additional evidence that the benefit of pre-resectional RFA cannot be explained solely on the basis of the cytoreductive effects of thermal ablation.

### Pre-resectional RFA augments circulating Th1/CD8^+^ T cell-directed cytokines and the ratio of CD8^+^ T cells to regulatory T cells in the tumor microenvironment

**Fig 3:**
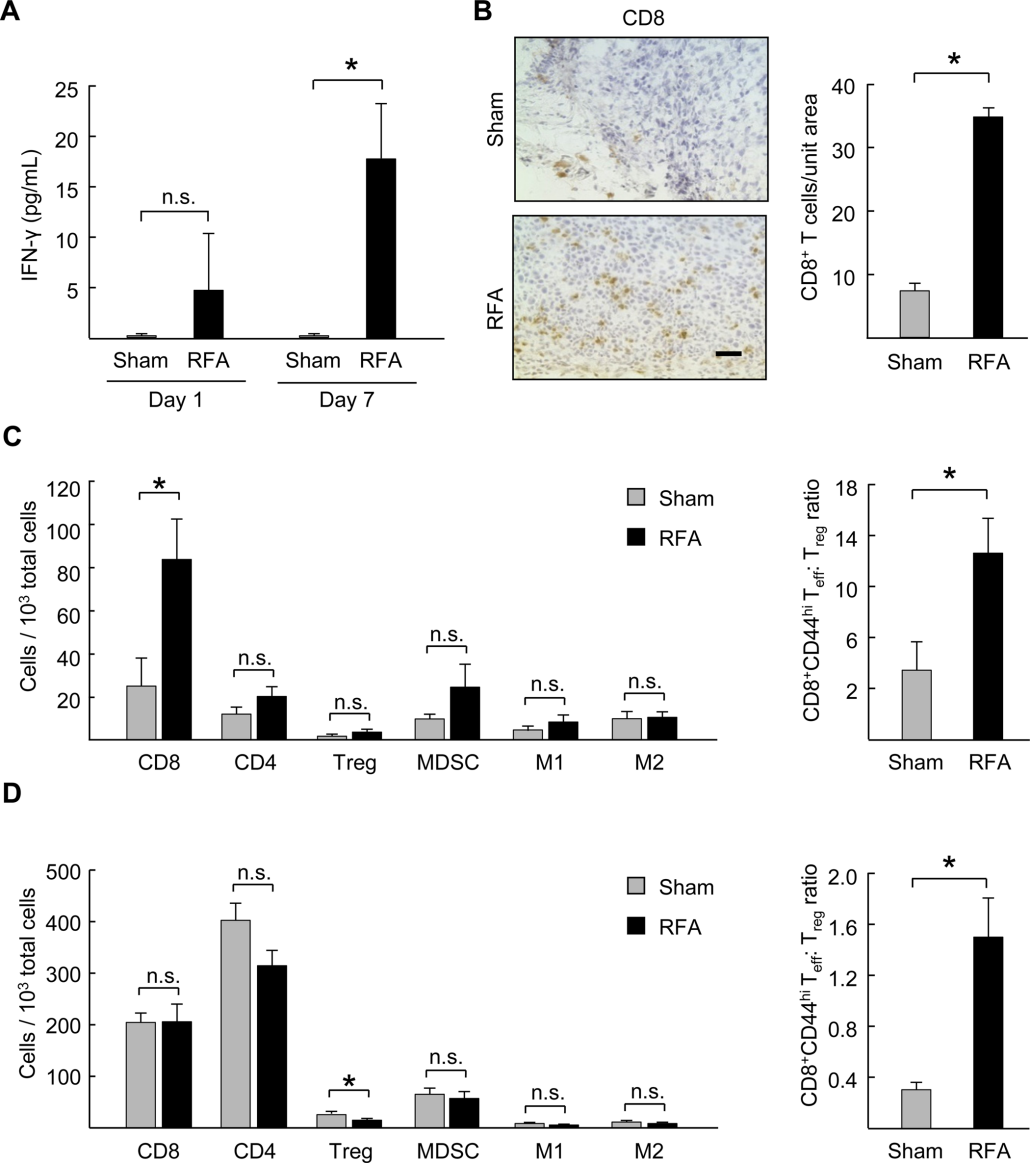
RFA increases circulating Th1/CD8-directed cytokines and ratio of CD8^+^ T cells to regulatory T cells in the tumor microenvironment. **(A)** IFN-γ profile in serum of CT26 tumor-bearing mice at 1 and 7 days post-RFA treatment. *P < 0.02, sham versus RFA. (B) Representative photomicrographs of immunostained endogenous CD8^+^ T cells and quantification of CD8^+^ T cell infiltration in tumor tissue sections 7 days after sham surgery or RFA. Scale bar, 100 μm. **(C)** *Left panel*, quantification of infiltrating leukocytes in tumors 7 days after treatment by flow cytometry; CD8^+^ T cells (CD3^+^CD8^+^), CD4^+^ T cells (CD3^+^CD4^+^), regulatory T cells (CD3^+^CD4^+^Foxp3^+^CD25^+^ T_reg_), myeloid-derived suppressor cells (CD11b^+^Gr-1^+^F4/80^-^); M1 (CD11b^+^Gr-1^-^F4/80^+^CD206^lo^MHCII^hi^) and M2 (CD11b^+^Gr-1^-^F4/80^+^CD206^hi^MHCII^lo^) macrophages. Data are for 10^3^ total cells in tumor specimens. *Right panel*, ratio of CD8^+^ effector T cells (T_eff_, L-selectin^low^ CD44^hi^): T_reg_ in tumors determined by flow cytometry. **(D)** *Left panel*, quantification of total leukocytes in spleens of tumor-bearing mice at 7 days post RFA treatment by flow cytometry. *Right panel*, ratio of CD8^+^ effector T cells (T_eff_, L-selectin^low^ CD44^hi^): T_reg_ in spleen. Data (**B-D**) are representative of ≥ 2 independent experiments (*n* = 3–5 mice per group); tumor volumes ranged from 1,100–1,700 mm^3^) at the time of RFA treatment. *P <0.05, sham versus RFA; n.s., not significant.

To gain insight into the role of the immune response in mediating the antitumor activity of pre-resectional RFA we assessed circulating immune cytokine levels after administration of RFA in CT26 tumors. We found a trend toward an increase in circulating concentrations of the prototypical Th1/CD8^+^ T cell-directed cytokine IFN-γ as early as 1 day post-RFA when compared with sham-treated mice and even stronger IFN-γ induction was evident 7 days after RFA treatment (Fig 3A). These data are in line with contributions of Th1/CD8^+^ T cell-directed cytokines in generating a systemic antitumor immune response.

These findings, together with evidence of long-term tumor control after combined RFA and surgery, led us to hypothesize that the enhanced local tumor control might involve changes in the proportion of intratumoral T cells subsets which has not been previously investigated following RFA. Preclinical studies in rabbits have reported increased intratumoral infiltration by CD3^+^ T cells in treated lesions after RFA although specific CD4^+^ or CD8^+^ T cell subsets were not examined [14, 27]. Clinical studies have additionally shown an increase in circulating tumor-specific CD4^+^ and CD8^+^ T cells in hepatocellular carcinoma patients (HCC) at 4 weeks post-RFA treatment [15]. Conversely, RFA reportedly causes a decrease in circulating CD4^+^ CD25^+^ Foxp3^+^ immunosuppressive regulatory T cells (T_reg_) 30 days post treatment in lung cancer patients which is noteworthy since T_reg_ counteract the function of cytotoxic CD8^+^ effector T cells [6, 28]. Moreover, high circulating levels of suppressive immune cells including T_reg_ or myeloid-derived suppressor cells (MDSC) have recently been shown to be prognostic of tumor recurrence after RFA in non-small-cell lung cancer (NSCLC) patients and HCC patients [29, 30]. However, the implications of these results have remained unclear since fluctuations in circulating immune cells are not necessarily reflective of their level of intratumoral localization after RFA.

To determine whether pre-resectional RFA affected intratumoral infiltration by specific immune cell subsets at the time of surgical excision, we quantified CD8^+^ T cells 7 days after ablation in resected CT26 tumors as well as in the spleen as an example of a peripheral lymphoid organ which is a site of immune activation. Immunohistochemical analysis showed minimal baseline intratumoral infiltration by endogenous CD8^+^ T cells in sham-treated mice (Fig 3B), in line with reports from our group and others that CT26 tumors are not permissive to intensive T cell accumulation [21, 22, 31]. However, a marked increase in intratumoral CD8^+^ T cell infiltration was detected after RFA treatment when compared to sham controls (Fig 3B). Flow cytometric analysis further revealed that infiltration by CD8^+^ T cells relative to the total intratumoral cell population was significantly improved 7 days after RFA whereas the relative density of total CD4^+^ T cells or the CD4^+^ CD25^+^ Foxp3^+^ T_reg_ subset remained unchanged (Fig 3C).

We examined the impact of RFA on CD8^+^ T cell:T_reg_ ratios within tumors since this is reportedly a strong prognostic indicator of a favorable outcome in metastatic colorectal cancer [32]. RFA was found to increase the overall ratio of CD8^+^ effector (L-selectin^low^ CD44^hi^) T cell:T_reg_ within RFA treated tumors (Fig 3C). A significant increase in the CD8^+^ T cell:Treg ratio was also observed in the spleen 7 days post-RFA (Fig 3D); however, this more favorable ratio was a consequence of an overall decrease in the proportion of T_reg_ within the splenic compartment rather than an increase in CD8^+^ T cells as was observed in the tumor microenvironment (Fig 3C). Decreased T_reg_ in the spleen could potentially reflect the reduced tumor burden that results from ablation therapy. The relative density of myeloid cells expressing phenotypic markers for CD11b^+^Gr1^+^ MDSC, CD11b^+^Gr-1^-^F4/80^+^CD206^lo^MHCII^hi^ M1 macrophages, or CD11b^+^Gr-1^-^F4/80^+^CD206^hi^MHCII^lo^ M2 macrophages subsets was unchanged by RFA treatment either within tumors or the spleen. (Fig 3C and 3D). Collectively, these results suggest that the RFA-induces changes in the immune contexture favoring Th1/CD8^+^ T cell activation within the tumor microenvironment and peripheral lymphoid organs contribute to improved local and systemic tumor control after surgical resection.

### Antigen-dependent expansion of CD8^+^ T cells occurs in tumor-draining lymph nodes and the tumor microenvironment following pre-resectional RFA

**Fig 4:**
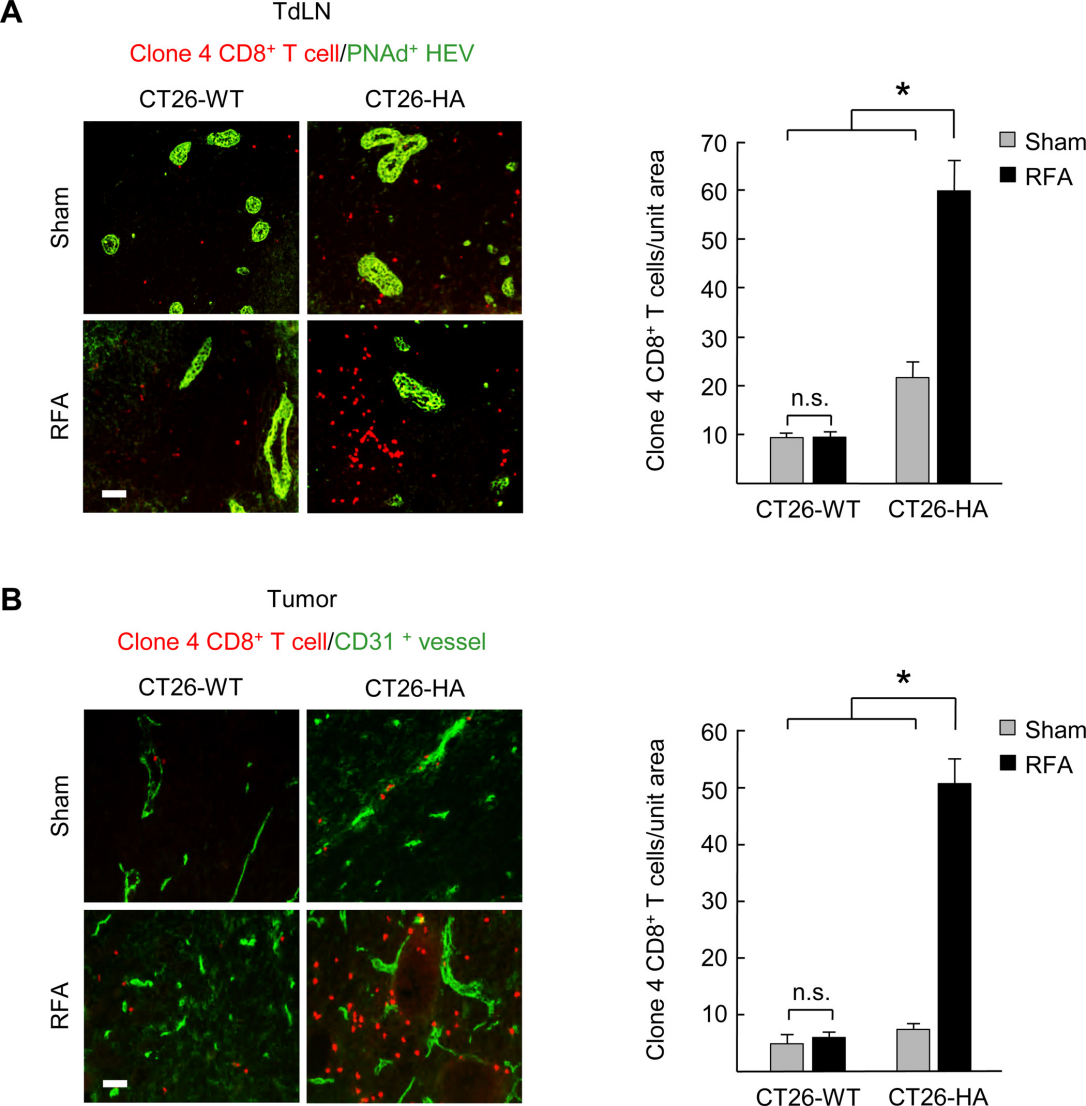
Antigen-dependent expansion of tumor-specific CD8^+^ T cell populations occurs in tumor-draining lymph nodes and the tumor microenvironment following pre-resectional RFA. **(A)** HA-specific Clone 4 naïve CD8^+^ T cells were adoptively transferred i.v. 6 hours after sham surgery or RFA treatment in mice bearing CT26-wild-type (WT) or CT26-HA tumors. Representative photomicrographs and quantification of TRITC-labeled Clone 4 CD8^+^ T cells (red) located outside PNAd^+^ HEV (green) in TdLN was determined 5 days after treatment with sham surgery or RFA. **(B)** Representative photomicrographs and quantification of TRITC-labeled Clone 4 CD8^+^ T cells (red) outside CD31^+^ vessels (green) in the non-ablated zone of CT26-WT or CT26-HA tumors at 7 days after treatment with sham surgery or RFA. Data are representative of two independent experiments (3 mice per group). Scale bars, 100 μm. *P < 0.001; n.s., not significant.

We reasoned that the increase in CD8^+^ effector T cells detected in tumors after RFA could result from the generation of an antigen-specific adaptive immune response, as reported previously [11, 14, 15], or alternatively from an increase in non-antigen-restricted localization due to the acute inflammation that ensues after the catastrophic cell death caused by thermal ablation. To distinguish between these possibilities, mice were implanted s.c. with murine CT26 colon adenocarcinoma engineered to express a surrogate tumor-associated antigen, hemagglutinin (CT26-HA), thus enabling us to monitor the tumor-antigen (HA)-specific CD8^**+**^ T cell response. A second group of mice was implanted with the parental CT26 tumor line lacking HA antigen. The tumor-specific CD8^+^ T cell response was tracked by intravenously transferring fluorescently-labeled naïve CD8^+^ T cells (L-selectin^hi^ CD44^low^) expressing HA-specific transgenic T cell receptors (derived from transgenic Clone 4 mice [21, 22]). Clone 4 T cells were transferred 6 hours after RFA and their tissue distribution was examined by immunofluorescence histology in TdLN (i.e., the initial site of naïve CD8^+^ T cell priming and expansion) and the tumor site (where CD8^+^ T cells execute antigen-restricted cytotoxic function) (Fig 4A and 4B). In order to rigorously quantify T cells located within the tissue parenchyma, fluorescent T cells were excluded from enumeration in TdLN if they were located within cuboidal high endothelial venules (HEV) that express peripheral lymph node address (PNAd) and are the major portals of naïve T cell entry [21]; transferred T cells were also excluded from quantification if they were within tumor vessels identified by staining for the CD31 pan-endothelial adhesion molecule [22, 24].

As expected from prior studies [22], during the first 24 hours after adoptive transfer naïve clone 4 CD8^+^ T cells preferentially seeded secondary lymphoid organs (LN, spleen) but were excluded from direct entry into tumors (data not shown). When the sentinel TdLN (i.e., ipsilateral inguinal node) was examined 5 days after transfer, we found that pre-resectional RFA caused a substantial increase in the number of HA-specific CD8^+^ T cells present within TdLN of CT26-HA tumor-bearing mice compared to sham controls (Fig 4A). Notably, this enhancement only occurred in response to cognate antigen, as evidenced by the failure of RFA to cause a significant increase in HA-specific T cells in TdLN of mice implanted with parental CT26 tumors (Fig 4A). When tumor tissues were evaluated 7 days after RFA (i.e., the same time-point as when we performed excisional surgery; Fig 1), we detected a profound increase in fluorescently-labeled HA-specific CD8^+^ T cells localized within CT26-HA tumors compared to parental CT26 tumors (Fig 4B). These findings establish that pre-resectional RFA impacts both the priming and effector phases of tumor-specific adaptive immune responses that are operative when excisional surgery was performed.

### Pre-resectional RFA elicits CD8^+^ T cell-mediated antitumor immunity in distant tumors

**Fig 5:**
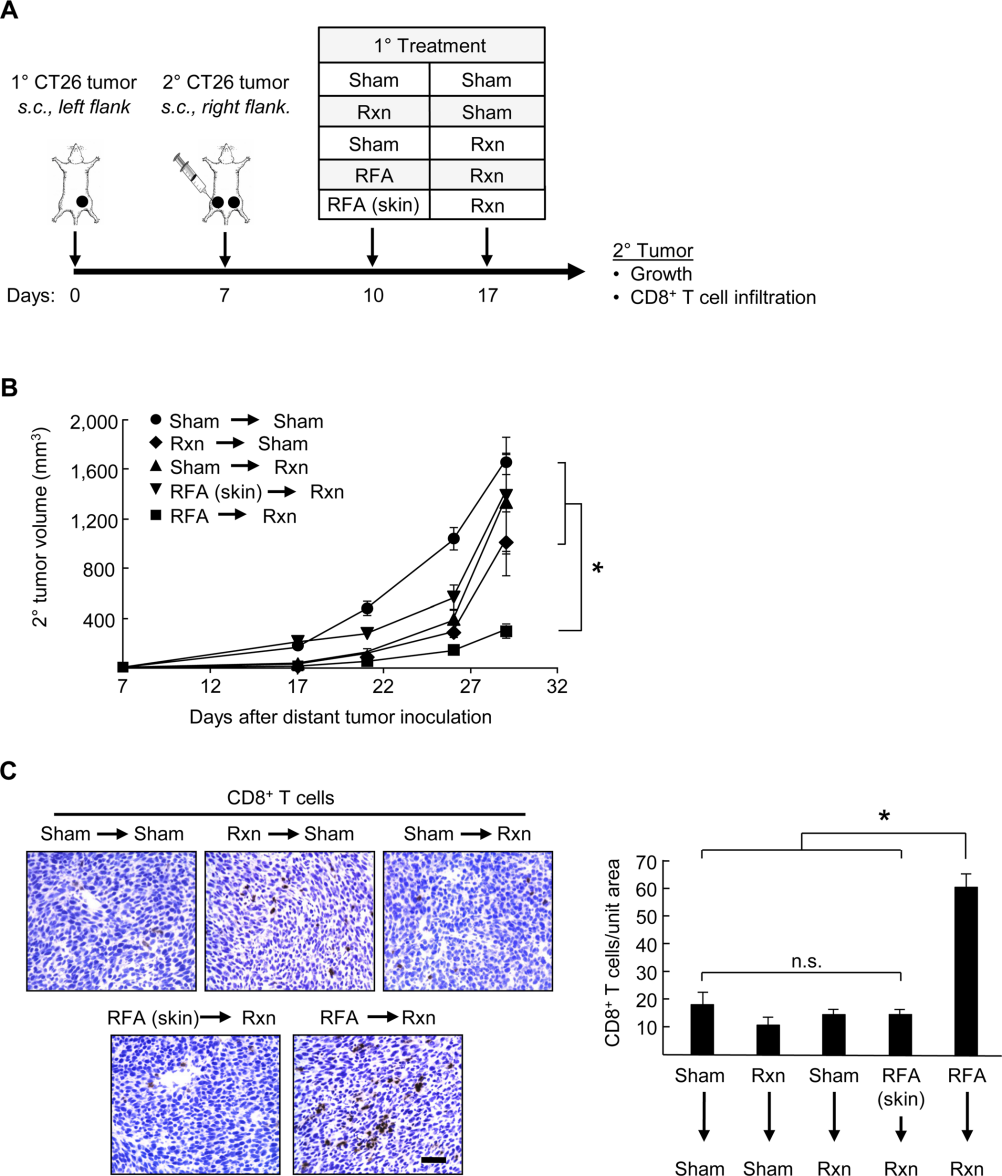
Pre-resectional RFA elicits systemic antitumor effects in distant tumors. **(A)** Time schedule outlining different treatments used in the experiements. BALB/c mice (5 mice/group) were injected s.c. with 10^6^ CT26 cells into the left flank on day 0 to establish primary tumor, followed by s.c. injection of 10^6^ CT26 cells in the right flank on day 7 to establish secondary tumor. Primary tumors or skin (at region outside of primary tumor; upper back) were treated as indicated with sham surgery, resection (Rxn), or RFA on days 10 or 17. **(B)** Growth curves of secondary CT26 tumors implanted s.c. on contralateral flank. *P <0.05. **(C)** Representative photomicrographs and quantification of immunostained endogenous CD8^+^ T cells in distant secondary s.c. CT26 tumors. *P < 0.001; scale bar, 100 μm.

**Fig 6:**
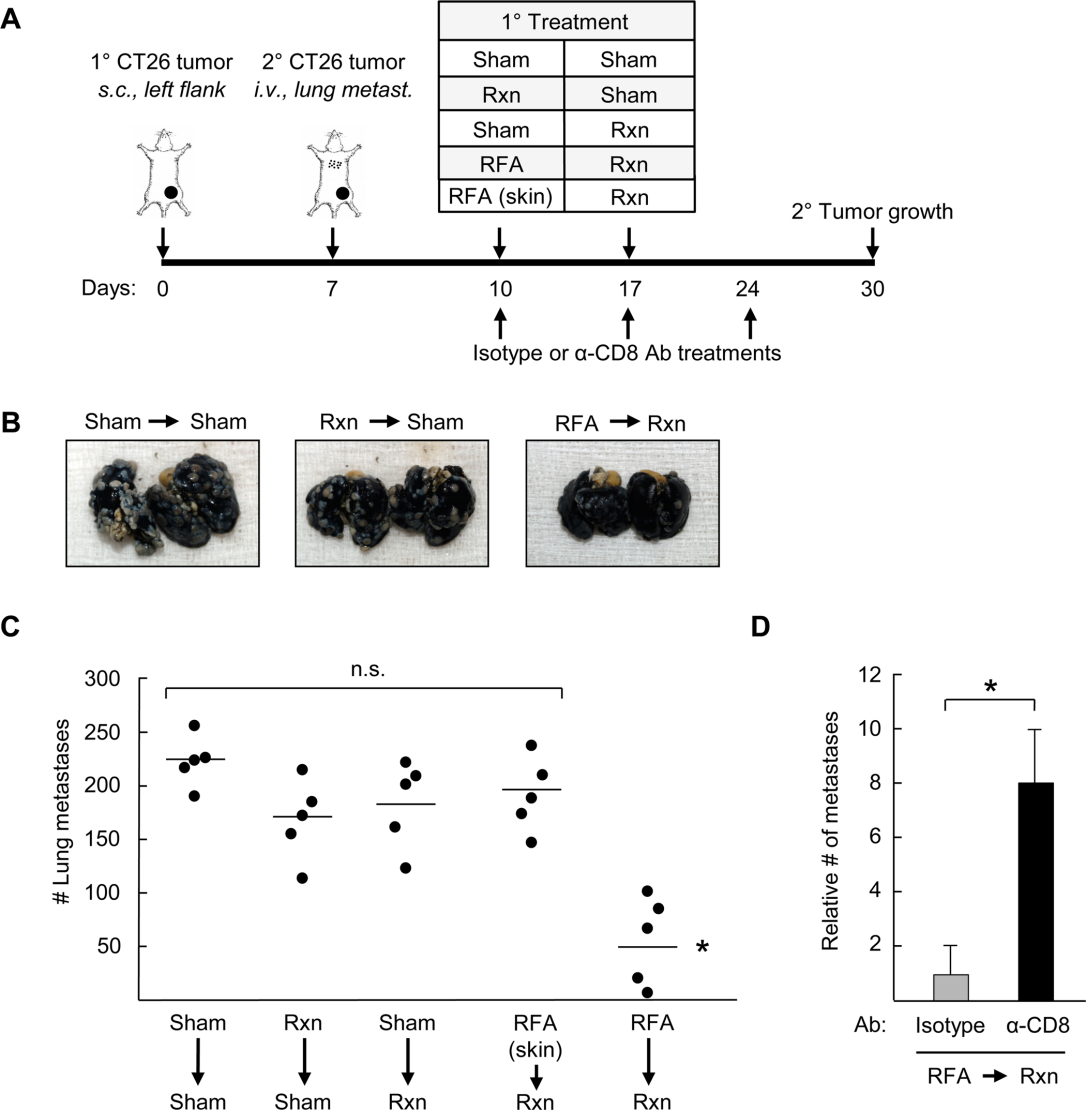
Therapeutic benefit of pre-resectional RFA in distant tumors depends on CD8^+^ T cell immunity. **(A)** Time schedule outlining different treatments used in the experiements. BALB/c mice (5 mice/group) were injected s.c. with 10^6^ CT26 cells into the left flank on day 0 to establish primary tumor, followed by injection of 10^5^ CT26 cells i.v. on day 7 to establish secondary tumors in the lungs. Primary tumors or skin (at region outside of primary tumor; upper back) were treated as indicated with sham surgery, resection (Rxn), or RFA on days 10 or 17. **(B)** Representative photographs of lungs harvested from mice that underwent the indicated treatments to their primary s.c. tumor. **(C)** Bars represent the average number of lung metastases. **(B-C)**, Data are representative of two independent experiments (*n* = 5 mice per group); n.s., not significant. *P <0.01 compared with all other groups. **(D)** Depleting α-CD8 antibody (Ab) or isotype antibody (400 ug/mouse) was injected i.p. every 7 days immediately post-RFA therapy. Lungs were harvested on day 30 and the number of metastases relative to the isotype control group was quantified. n = 3 mice per group. *P <0.05, sham versus RFA.

Abscopal effects attributed to systemic adaptive immunity were first observed after radiotherapy whereby treatment of a primary lesion led to widespread inhibition of tumor growth outside the treatment region [33]. Similar remote control of metastatic tumor progression has been reported clinically in cancer patients after treatment with RFA [16–18] and in rodent models of HCC [20]. These observations raised the question of whether pre-resectional RFA could elicit distant tumor control associated with CD8^+^ T cell activity in murine models of disseminated colorectal cancer. For these studies, primary CT26 tumors were established by s.c. innoculation and 7 days later, secondary tumors were introduced at distant sites by s.c. injection in the contralateral flank (Fig 5) or through i.v. injection in an orthotopic model of end-stage colorectal metastasis in the lungs (Fig 6). The primary tumor was treated with RFA on day 10 and surgically excised on day 17 in order to monitor distant tumor growth. Control groups included sham surgeries (days 10 and/or 17), or resection coupled with sham surgery (performed on days 10 or day 17) (Figs 5A and 6A). We also included a control group in which RFA was administered on day 10 in the normal skin (i.e., outside the region of the primary tumor, upper back) followed by resection of primary tumor on day 17, in order to investigate whether acute inflammation unrelated to tumor-antigen specific stimulation has an impact on secondary tumor growth.

Results in Fig 5 and Fig 6 demonstrate that pre-resectional RFA was the only treatment group to significantly improve tumor control at distant sites. Specifically, RFA treatment of primary tumors followed by surgical resection markedly limited outgrowth of secondary tumors at contralateral s.c. sites which was associated with enhanced CD8^+^ T cell infiltration in distant tumors (Fig 5B and 5C). Identical CD8^+^ T cell infiltration was observed regardless of whether surgery was performed after RFA *(data not shown)*. Surgical excision alone had no impact on CD8^**+**^ T cell infiltration in distant tumors (Fig 5C), definitively establishing resection as a non-immunogenic treatment modality in these murine models of distant colorectal tumor growth. Pre-resectional RFA also limited the number of metastatic nodules detected in the lung >2 weeks after ablation of the primary tumor (Fig 6B and 6C). Acute inflammation resulting from RFA treatment of normal skin did not affect secondary tumor growth at contralateral sites or in the lung (Fig 5B and Fig 6C).

**Fig 7:**
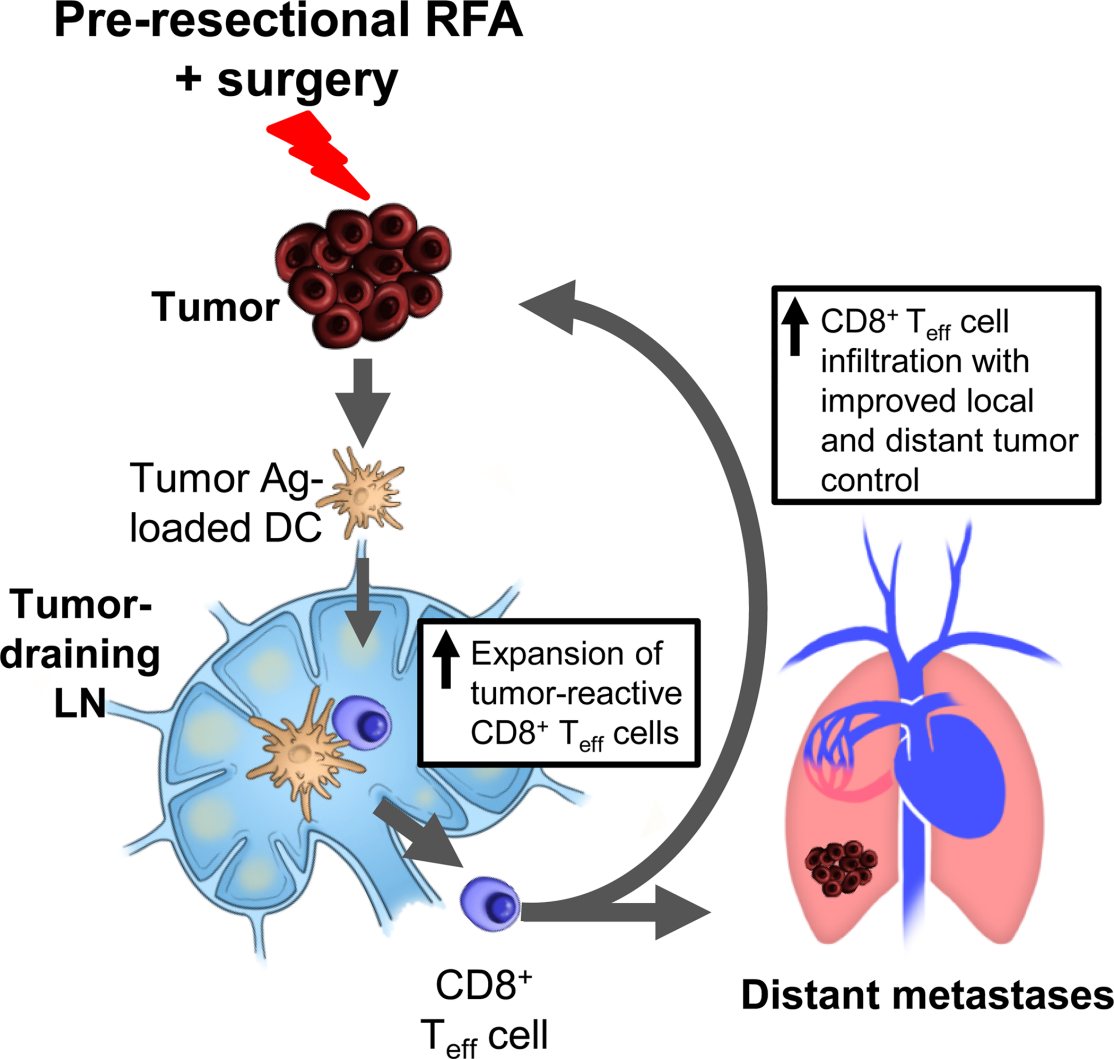
Proposed model for induction of systemic CD8^+^ T cell-mediated local and systemic tumor control by pre-resectional RFA. Pre-resectional RFA reportedly promotes delivery of tumor-antigen (Ag)-loaded antigen-presenting cells such as dendritic cells (DC) to tumor-draining lymph nodes. This, in turn, is proposed to result in activation and expansion of CD8^+^ effector T cells (T_eff_) which execute cytotoxic functions leading to improved local and distant tumor control after excisional surgery.

To directly interrogate whether the distant tumor control induced by pre-resectional RFA was due to systemic adaptive immunity, we depleted CD8^+^ T cells by antibody treatment beginning at the time of RFA treatment and evaluated the impact on lung tumor outgrowth compared with the isotype antibody-treated control group (Fig 6A and 6D). Antibody-mediated depletion of CD8^+^ T cells was confirmed by flow cytometry whereby CD8^+^ T cells comprised < 2% of circulating CD3^+^ T cells compared to >18% in the isotype control antibody-treated group at the time-point when lungs were analyzed for tumor burden (i.e., 30 days after primary tumor implantation; S3 Fig). The data in Fig 6D show that the therapeutic benefit of pre-resectional RFA was abrogated in the absence of the CD8^+^ T cell arm, as indicated by the significant increase in lung tumor nodules detected upon CD8^+^ T cell depletion relative to isotype antibody-treated controls. Collectively, our results support a model in which pre-resectional RFA stimulates CD8^+^ T cell adaptive immunity in TdLN which, in turn, mediates tumor control at local and distant tumor sites (Fig 7).

## Discussion

Local and distant recurrence remains a common problem after surgery with curative-intent for solid malignancies despite advances in surgical technique, cytotoxic chemotherapy, and molecular-targeted therapy. For example, the incidence of recurrence is up to 60% in resected patients with colorectal cancer liver metastasis [1–3], 70–80% for HCC [34, 35] and 50–70% for cholangiocarcinoma [36, 37]. The pathological stage, resection margin status, tumor burden (i.e., size and number of lesions), and ‘immunoscore’ based on the type of immune infiltrates, have each been identified as prognostic indicators of cancer relapse after resection [5, 7]. The importance of immunoscore indicates that the status of the antitumor adaptive immune response before surgical resection is a crucial determinant for predicting prognosis and disease-free survival [5, 7]. However, immune surveillance is rarely sufficient to prevent tumor progression and sparse intratumoral CD8^**+**^ T cell infiltration at the time of surgery is correlated with poor prognosis in a majority of patients [7]. The current study demonstrates that outcome, with respect to local recurrence and overall survival in aggressive murine models of colon adenocarcinoma or melanoma, is significantly improved when RFA is administered 1 week prior to excisional surgery. A central finding was that the abscopal benefit of pre-resectional RFA in distant tumors was abrogated in immunocompetent mice following CD8^+^ T cell depletion, thereby definitively establishing the requirement for systemic CD8^+^ T cell-immunity for the antitumor activity of RFA in a neoadjuvant setting prior to surgery.

According to the immunoediting hypothesis, tumor progression occurs as the immune system becomes tolerized or immune-resistant tumor variants evade immune detection [4, 38]. Our results, together with prior studies suggest that the inflammatory effects of RFA can break the cycle of immune evasion by creating a substantial *in situ* source of acute inflammatory signals and tumor antigens in the form of necrotic tumor cells and cellular debris which have the potential to generate systemic immunity [8]. Here, we provide evidence that pre-resectional RFA causes a profound increase in CD8^+^ effector T cell infiltration at the residual tumor site which is indicative of activation of the innate and/or adaptive immune system. These findings extend prior observations that RFA stimulates a marked local inflammatory response with dense CD3^+^ T cell infiltration in patient tumors and animal tumor models [14, 15, 20].

The observed increase in CD8^+^ effector T cells at tumor sites is predicted to provide a therapeutic benefit given evidence that high CD8^+^ T cell infiltration is a positive prognostic indicator in patients with primary colorectal cancer and colorectal liver metastases [5, 7]. T_reg_ appear to have a complex relationship with colorectal cancer [7, 39] and our results for this T cell subset must be interpreted in the context of a small animal model where RFA is administered in s.c. tumors. High intratumoral T_reg_ infiltration is a positive prognostic indicator in primary colorectal cancer patients where this T cell subset is thought to suppress chronic inflammation induced by local enteric bacteria that leads to cancer initiation [7, 39–43]. Conversely, T_reg_ appear to contribute to immune evasion in patients with colorectal liver metastasis since a high number of T_reg_ relative to CD8^+^ T cells correlates with a poor prognosis [32]. Thus, our findings showing that RFA increases CD8^+^ effector T cell:T_reg_ ratios in colorectal tumors located outside the colon as well as in peripheral lymphoid organs are suggestive of a more favorable microenvironment where immune activation as well as the cytolytic activity of CD8^+^ effector T cells is not countermanded by T_reg_. While recent studies in NSCLC and HCC patients have shown that circulating MDSC burden inversely correlates with the probability of local recurrence after RFA [29, 30], we did not observe an acute effect of RFA (i.e., within 1 week) on MDSC localization at tumors or peripheral lymphoid organs (spleen). However, our studies do not exclude the possibility that myeloid cell populations become altered during tumor progression at later time-points.

Our findings further demonstrate that pre-resectional RFA increases tumor-specific CD8^+^ T cells in TdLN. These observations are likely related to the increase in CD8^+^ T cells detected in the circulation of patients [15] as well as within tumors after pre-resectional RFA as described in the present study. These results expand on current understanding of the tightly integrated mechanisms underlying RFA stimulation of adaptive immunity. Ablation-induced immunogenic death of tumor cells is known to generate proinflammatory signals such as heat shock protein 70 and gp96 [44–46] which, in turn, induce activation and maturation of DC and their migration to draining lymph nodes [11, 47–49]. Additionally, increased DC-mediated cross-priming of tumor-antigen restricted naïve CD8^+^ T cells within TdLN occurs in response to RFA and is accompanied by proliferative expansion of the tumor-reactive CD8^+^ effector T cell pool [13, 26]. The robust increase in tumor-specific CD8^+^ T cells detected in TdLN in the current study is particularly notable given that TdLN represent a strongly immunosuppressed microenvironment due to local T_reg_ and tolerogenic DC [50]. Evidence that delayed growth at distant tumor sites depends on CD8^+^ T cells following pre-resectional RFA is further in line with clinical observations of systemic tumor control after RFA treatment in renal cell carcinoma and prostate cancer patients [16–18].

The current study supports a scenario in which the immunostimulatory activity of RFA could be exploited clinically in a neoadjuvant setting prior to surgery in patients with high risk of local and distant recurrence. There is already precedent for neoadjuvant radiation therapy and chemotherapy which are standard-of-care before surgical resection for patients with locally-advanced tumor with a high-risk of recurrence. Although these treatments are effective in some types of solid malignant tumors and induce systemic antitumor immunity [33, 51], their efficacy is limited by a number of parameters including the oxygenation status of the tumor, variations in the inherent sensitivity of tumors cells to chemotherapy or radiation, and acquired chemo- and radio-resistance [52]. Thus, thermal ablation techniques such as RFA offer an advantage since they are effective in a wide variety of tumors regardless of tumor sensitivity to radiation therapy or chemotherapy.

The fact that RFA can be performed with minimally invasive technique under radiological guidance without general anesthesia, and that it takes only a week to induce tumor-specific systemic immunity, suggests that pre-resectional RFA will not delay or complicate subsequent definitive excisional surgery. This represents an additional benefit over neoadjuvant chemotherapy or radiotherapy which must be administered over several weeks-to-months to reduced tumor burdens before surgical resection. Indeed, intraoperative RFA followed immediately by resection has already been reported for early stage breast cancer patients [53–55] and in one study, RFA was performed 1–3 week prior to surgery in breast cancer patients [55]. The primary objective of these clinical reports was to evaluate the impact of RFA on coagulative necrosis of local tumor cells so immunological endpoints were not investigated; however, they provide proof-of-concept that pre-resectional RFA is feasible and can be safely applied in a clinical setting.

In summary, our results demonstrate that pre-resectional RFA is an effective immune adjuvant that improves local tumor control and overall survival in a lethal murine model of colorectal cancer. The absence of immune adjuvant effect of pre-resectional RFA in immunodeficient mice or in CD8^+^ T cell-depleted mice reveals the involvement of systemic adaptive immunity in this immune-adjuvant ablation modality. In addition, our study provides insight into the contributions of the immune system to the systemic tumor control observed in patients after RFA. It is important to note that despite the benefit of RFA in a neoadjuvant setting prior to surgery shown in the current report, this modality was not universally protective and tumor progression was observed in a subset of mice that received combination therapy. These data raise the possibility that further benefit might be conferred if the CD8^+^ T cell arm of anti-tumor immunity was boosted by combining neoadjuvant RFA with immunotherapeutics. Noteworthy, in this regard are preclinical reports showing that the systemic antitumor immunity induced by RFA can be enhanced by co-administration of immunological modifiers such as cytotoxic T-lymphocyte-associated protein 4 (CTLA-4) blockade [11, 13]; T_reg_ depletion [13], IL-2 [23, 56]; or by administration of IL-7 and IL-15 [8], macrophage inflammatory protein-1α [57], heat-shocked tumor cell lysate-pulsed dendritic cells [45], poxviral vaccines [19], or CpG-oligodeoxynucleotides [26, 27]. While colon cancer patients are not generally responsive to targeting of the programmed death-1 (PD-1)/PD-1 ligand (PD-L1) axis [58, 59], this immunosuppressive pathway has been implicated in blunting CD8^+^ T cell antitumor immunity in CT26 colorectal cancer [60, 61] and in clinical trials in a subset of colorectal cancer patients with mismatch-repair deficiency [62, 63]. Thus, the use of pre-resectional RFA in combination with antagonists of the PD-1/PD-L1 axis is another promising avenue for future investigation for patients with high risk of local and distant recurrence.

## Supporting Information

S1 Fig Pre-resectional RFA improves local CT26 tumor control and survival. **(A)** Time schedule outlining different treatment groups. BALB/c mice (5 mice/group) were implanted subcutaneously with 10^6^ CT26 cells into the left flank on day 0. Treatments at the time-points indicated included sham surgery (in contralateral flank), sham RFA (probe inserted into tumor without current), resection (Rxn), partial Rxn (~50% of tumor excised), or RFA. Tumor growth curves **(B)**, and survival curves **(C)** of mice bearing CT26 tumors in different treatment groups. In **(B)**, the number of long-term survivors without tumor recurrence detected at 100 days is indicated for each experimental group. Data are representative of two independent experiments. *P <0.05, RFA + Rxn compared to the indicated groups; **P = 0.072 for RFA + Rxn compared to Rxn + sham (Rxn) group.
(TIF)

S2 Fig Therapeutic benefit of pre-resectional RFA in B16 murine melanoma. **(A)** Time schedule outlining different treatment groups. C57BL/c mice (5 mice/group) were implanted subcutaneously with 3x10^5^ B16.F10 cells into the left flank on day 0. Treatments at the time-points indicated included sham surgery (in contralateral flank), resection (Rxn), or RFA. Tumor growth curves **(B)** and survival curves **(C)** of mice are shown for different treatment groups. In **(B)**, the number of long-term survivors without tumor recurrence at 150 days is indicated for each experimental group. Data are representative of two independent experiments. *P <0.05, RFA + Rxn compared to the other groups determined Gehan-Breslow-Wilcoxon test.
(TIF)

S3 Fig CD8^+^ T cell depletion in vivo following anti-CD8 antibody administration. Anti-CD8α antibody was used to deplete CD8 T cells systemically. Peripheral blood leukocyte populations were monitored weekly to determine the extent of depletion. Representative flow cytometry dot plots show CD8β^+^ T cell depletion 30 days after primary tumor cell implantation; i.e., just before lungs were harvested for quantification of metastatic nodules (see outline for experimental design in Fig 6A). Percentages shown are of total CD3^+^ T cell population. Data in bar graphs are for n = 3 mice per treatment group; * *P* = 0.0002.
(TIF)
