# Supplementary figures and images for: Immune Adjuvant Activity of Pre-Resectional Radiofrequency Ablation Protects against Local and Systemic Recurrence in Aggressive Murine Colorectal Cancer
Source: PLoS One. 2015 Nov 23;10(11):e0143370. doi: 10.1371/journal.pone.0143370 (PMC4657935; doi:10.1371/journal.pone.0143370)

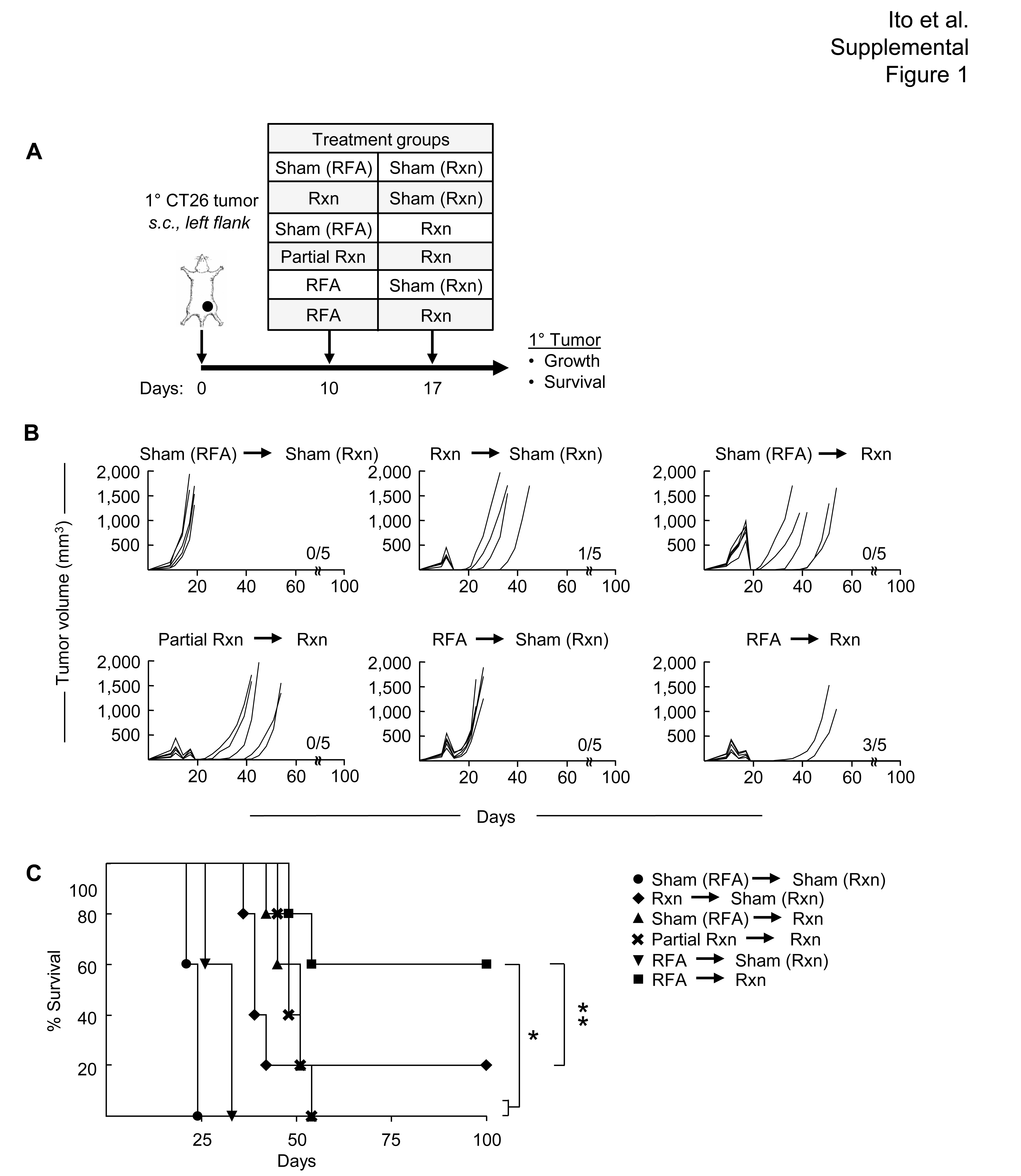

Supplement: S1 Fig — (A) Time schedule outlining different treatment groups. BALB/c mice (5 mice/group) were implanted subcutaneously with 106 CT26 cells into the left flank on day 0. Treatments at the time-points indicated included sham surgery (in contralateral flank), sham RFA (probe inserted into tumor without current), resection (Rxn), partial Rxn (~50% of tumor excised), or RFA. Tumor growth curves (B), and survival curves (C) of mice bearing CT26 tumors in different treatment groups. In (B), the number of long-term survivors without tumor recurrence detected at 100 days is indicated for each experimental group. Data are representative of two independent experiments. *P <0.05, RFA + Rxn compared to the indicated groups; **P = 0.072 for RFA + Rxn compared to Rxn + sham (Rxn) group. (TIF) [file pone.0143370.s001.tif]

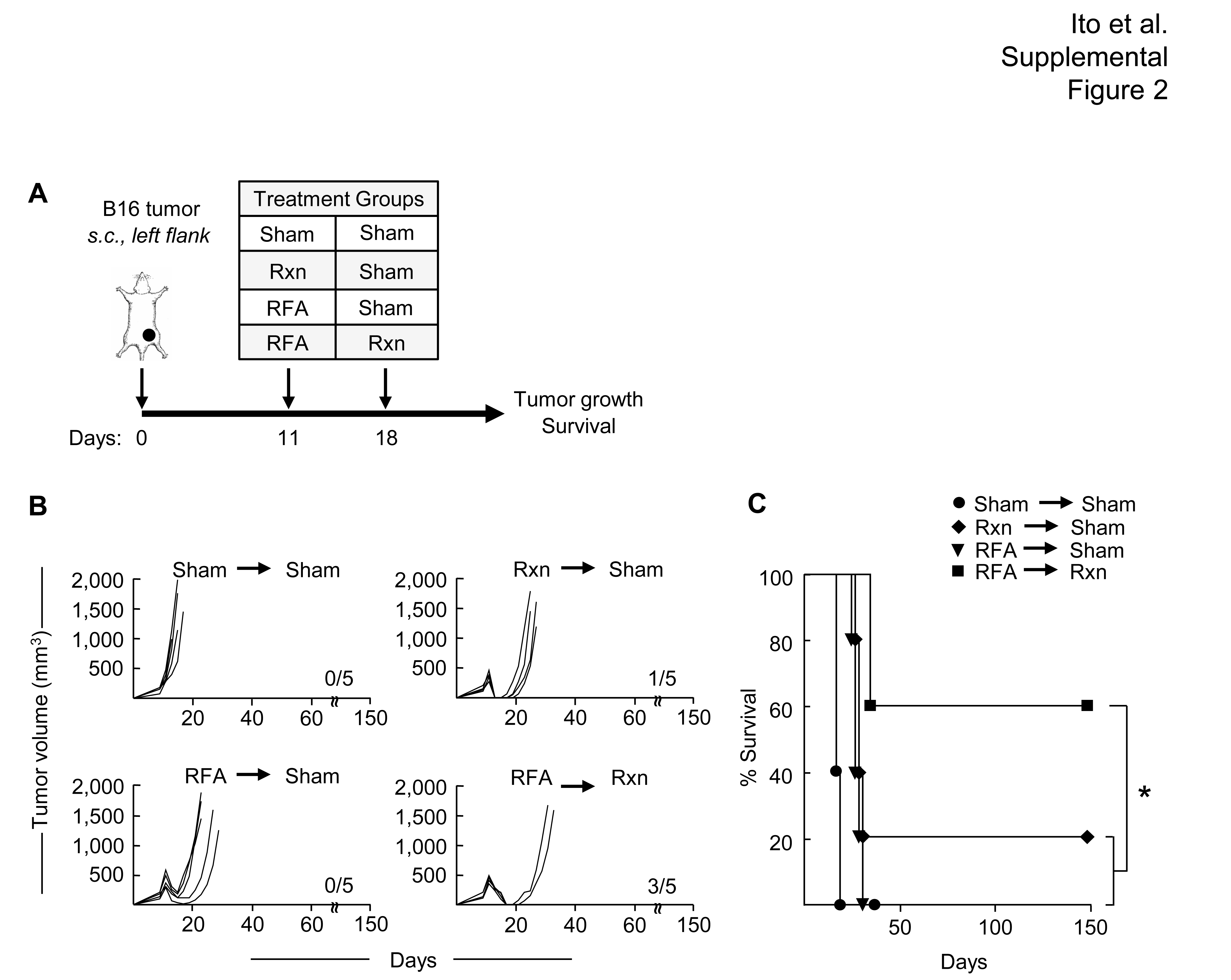

Supplement: S2 Fig — (A) Time schedule outlining different treatment groups. C57BL/c mice (5 mice/group) were implanted subcutaneously with 3x10^5^ B16.F10 cells into the left flank on day 0. Treatments at the time-points indicated included sham surgery (in contralateral flank), resection (Rxn), or RFA. Tumor growth curves (B) and survival curves (C) of mice are shown for different treatment groups. In (B), the number of long-term survivors without tumor recurrence at 150 days is indicated for each experimental group. Data are representative of two independent experiments. *P <0.05, RFA + Rxn compared to the other groups determined Gehan-Breslow-Wilcoxon test. (TIF) [file pone.0143370.s002.tif]

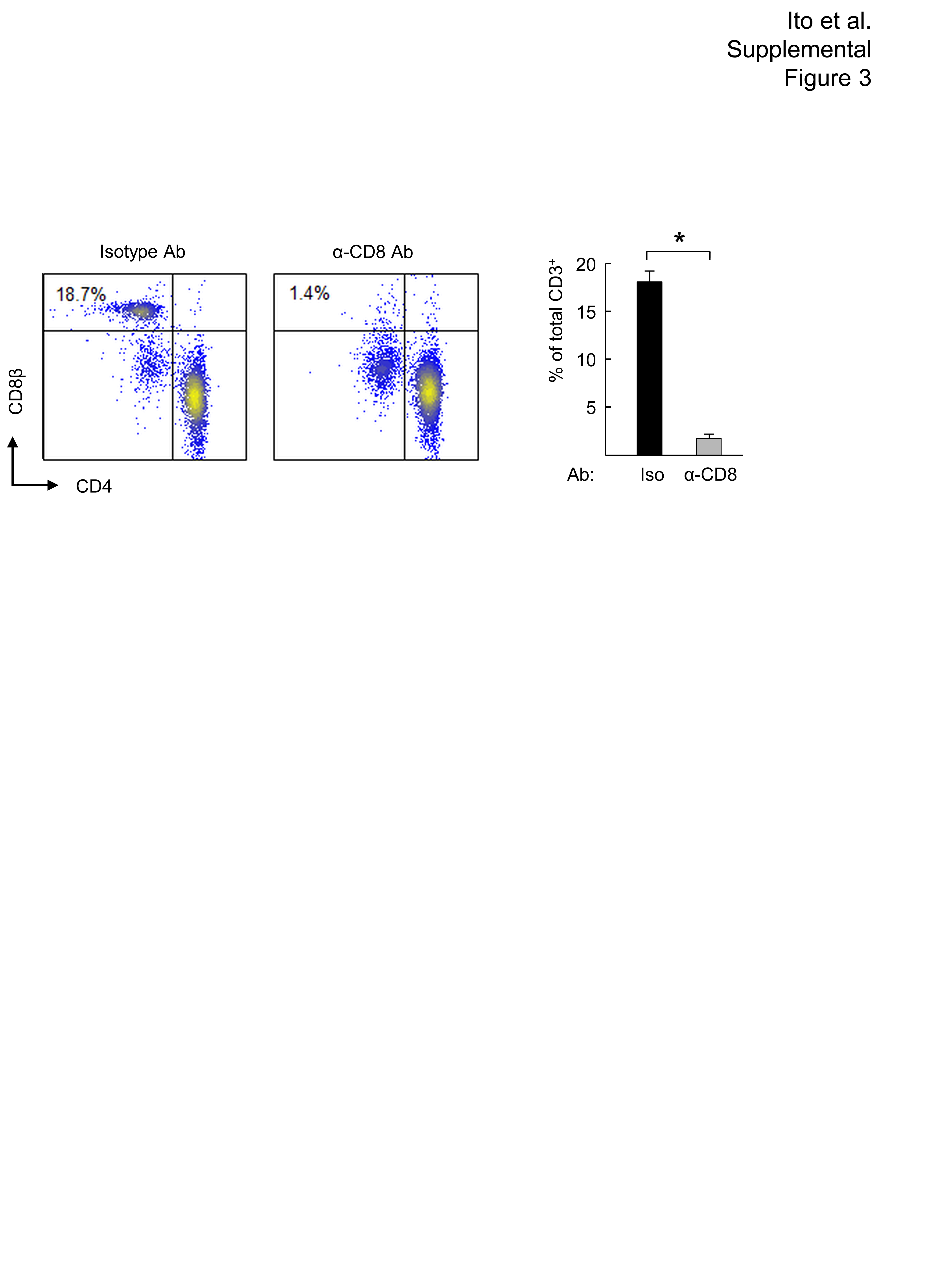

Supplement: S3 Fig — Anti-CD8α antibody was used to deplete CD8 T cells systemically. Peripheral blood leukocyte populations were monitored weekly to determine the extent of depletion. Representative flow cytometry dot plots show CD8β+ T cell depletion 30 days after primary tumor cell implantation; i.e., just before lungs were harvested for quantification of metastatic nodules (see outline for experimental design in Fig 6A). Percentages shown are of total CD3+ T cell population. Data in bar graphs are for n = 3 mice per treatment group; * P = 0.0002. (TIF) [file pone.0143370.s003.tif]
